# Optimization of ethanol production using newly isolated ethanologenic yeasts

**DOI:** 10.1016/j.bbrep.2020.100886

**Published:** 2021-01-07

**Authors:** Asmamaw Tesfaw, Ebru Toksoy Oner, Fassil Assefa

**Affiliations:** a Department of Biology, Debre Berhan University, P.O Box,445, Debre Berhan, Ethiopia; b, Department of Bioengineering, Marmara University, Goztepe Campus, P.O.Box 34722, Istanbul, Turkey; c Department of Microbial Cellular and Molecular Biology, Addis Ababa University, P.O Box,1176, Addis Ababa, Ethiopia

**Keywords:** *Candida humilis*, *Kluyveromyces marxianus*, *Pichia fermentans*, *Saccharomyces cerevisiae*, *Yeast*, *Ethanol production*

## Abstract

*Yeasts are important microorganisms used for ethanol production; however, they are not equally efficient in the amount of ethanol production under different environmental conditions. It is, therefore, necessary to screen for elite strains to utilize them for commercial production of these commodities. In this study, yeasts were isolated from different Ethiopian traditional fermented alcoholic beverages (teji, tella, shamiata and areqe tinisis), milk and ergo, teff and maize dough, soil and compost, flowers, and fruits to evaluate their potential use for ethanol fermentation process. Isolates were screened for efficient ethanol production and the selected ones were identified using phenotypic and genetic characters using D1/D2 region of LSU rDNA sequence analysis. The yeast isolates were evaluated based on their growth and fermentation of different carbon sources. Response surface methodology (RSM) was applied to optimize temperature,* pH *and incubation time using central composite design (CCD) in Design-Expert 7.0.0. A total of 211 yeasts colonies were isolated of which 60% were ethanologenic yeasts (ethanol producers) and 40% were non-ethanol producers. The yeast population detected from various sources was in the range of 10*^*5*^*CFU from traditional foods and beverages to that of 10*^*3*^*CFU from fruits and soil samples. The data also showed that the number of colony types (diversity) did not correlate with population density. The highly fermentative isolates were taxonomically characterized into four genera, of which 65% of the isolates (ETP37, ETP50; ETP53, ETP89, ETP94*) *were categorized under Saccharomyces cerevisiae*, *and the remaining were Pichia fermentans ETP22, Kluyveromyces marxianus ETP87, and Candida humilis ETP122. The S. cerevisiae isolates produced ethanol (7.6-9.*0 g/L*) similar with K. marxianus ETP87 producing 7.*97 g/L*; comparable to the ethanol produced from commercial baker's yeast (8.*43 g/L*) from* 20 g/L *dextrose; whereas C. humilis ETP122* and *P. fermentans ETP22 produced 5.*37 g/L *and 6.*43 g/L *ethanol, respectively. S. cerevisiae ETP53, K. marxianus ETP87, P. fermentans ETP22 and C. humilis ETP122 tolerated 10% extraneous ethanol but the percentage of ethanol tolerance considerably decreased upon 15%. S. cerevisiae ETP53 produced ethanol optimally at* pH *5.0, 60 h, and 34*^*o*^*C.* pH *4.8, temperature 36*^*o*^*C, and 65 h of time were optimal growth conditions of ethanol fermentation by K. marxianus ETP87. The ethanol fermentation conditions of P*. *fermentans ETP22 was similar to S. cerevisiae ETP53 though the ethanol titer of S. cerevisiae ETP53 was higher than P. fermentans ETP22. Therefore, S. cerevisiae ETP53, K. marxianus and P. fermentans ETP22 are good candidates for ethanol production*.

## Highlights

• Among 211 yeast isolates, 126 were able to ferment dextrose.
• *Saccharomyces cerevisiae*, *Pichia fermentans*, *Kluyveromyces marxianus*, and *Candida humilis* were isolated from traditional alcoholic drinks, compost, ergo, and enjera dough, respectively.
• *Kluyveromyces marxianus* ETP87 can be used to produce ethanol from whey.
• *Saccharomyces cerevisiae*, *Kluyveromyces marxianus* and *Pichia fermentans* are good candidate for ethanol production.

## 1 Introduction

Yeasts are cosmopolitan microorganisms that are mostly found in natural ecosystems rich in sugar [1]. Mohd Azhar et al. [2]) estimated that over 150, 0000 yeast species are distributed on earth of which only 1% is known.

Plants are the preferred habitats for yeasts and they include nectars, flowers, fruits, decaying tissues, and tree saps [3,4]. These plant parts also attract insects that not only help the distribution of yeasts to different habitats but also play a great role in diversifying the yeast communities in flowers making the yeast diversity seasonal since the pollinating insects are mostly seasonal [[4], [5], [6]]. In addition, yeasts are widely distributed in the soil [1].

Yeasts are among the essential microorganisms found in traditional fermented foods and beverages in Ethiopia. The yeasts that dominate the Ethiopian dairy products are *Kluyveromyces* (46.8%), *Sporobolomyces* (31.5%), *Candida* (12.5%), *Torulopsis* (6%) and *Leucosporidium* (3.2%) [[7], [19]]. This study also showed that *Candida milleri*, *Rhodotorula mucilaginosa*, *K. marxianus*, *Pichia naganishii*, *Rhodotorula glutinis*, *K. marxianus* and *Pichia membranefaciens* were isolated from staple fermented food enjera and kocho. Studies also showed that *S. cerevisiae*, *Kluyveromyces bulgaricus*, *Debaromyces phaffi* and *Kluyveromyces veronae* are found in *teji tella*, *shamita*, and *borde*[19]. This shows that the Ethiopian fermented drinks and food could be good sources of yeast for ethanol production. Most of the hitherto studies focused on the yeast profile of the different food and beverage sources. However, there is a dearth of information on the efficiency of these isolates for ethanol production.

Traditionally, yeasts from the genus *Saccharomyces* are used in bakery and brewery industries for ethanol production of high spirit and industrial grade ethanol for human consumption from simple sugars. However, simple sugars are relatively expensive substrates for economical ethanol production. On the other hand, ethanol can also be produced from agricultural crush (starch and lignocellulose), sugar industry wastes (sugar cane and beet molasses), and dairy industry waste (whey) with a dual purpose of producing energy from cheap sources and alleviation of environmental pollution [9]. However, the uses of different agricultural wastes require the selection of yeasts that are capable of utilizing substrates derived from hydrolysis of complex carbohydrates.

It is well established that yeast growth and ethanol production are influenced by different nutritional and environmental factors such as temperature, pH, oxygen, and initial sugar concentrations [10,12]. Temperature and pH affect membrane turgidity, enzymatic activity and metabolism of yeast cells [13]. They usually prefer acidic pH that enables to control bacterial growth at industrial level. Consequently, yeasts which are active and tolerant to high temperature and low pH are ideal for industrial bioethanol production.

Yeasts also require different nutrients of which sugar mostly limit their growth and activity. According to Ref. [10]; initial sugar concentration reduces the average specific growth rate of yeasts but enhances their substrate uptake. For this reason, yeast osmo-tolerance at the beginning of fermentation and ethanol-tolerance in late fermentation is the pre-requisite for very high gravity (VHG). [14] showed that high sugar concentration commonly greater than 200 g/L produced high ethanol titer from VHG fermentation. This requires the evaluation of glucose concentration on yeast isolates for their potential for VHG ethanol fermentation.

Ethanol also inhibits yeast growth and cell viability. It affects various transport systems, inhibits glycolytic enzymes, damages mitochondrial DNA, modifies the fluidity of plasma membrane, lowers RNA, denatures proteins and stimulates ATPase activity [12,15]. Higher ethanol reduces nutrient uptake in yeast [12]. Apart from that, environmental stresses reduce yeast tolerance to ethanol [16]. Yeast which is tolerant to ethanol is a prerequisite for high fermentation efficiency and high ethanol yield [17]).

Optimization of multiple variables using conventional strategies like one factor at a time, i.e. carrying out many experiments separately, cannot evaluate the interactions among different variables and hence one cannot derive statistical conclusions regarding alternative effects between components. Recently, statistical experimental methods are employed using mathematical models in bioprocesses [13].

Among these methods, response surface methodology (RSM) is suitable for optimization in different disciplines [13,18]. It enables to design experiments, build models, evaluate interactions, look for optimum conditions for responses, and reduce the number of experiments [13]. This method has been used to optimize various chemical production including bioethanol production [18].

The aim of this research was to isolate, characterize and identify ethanologenic yeasts and optimize growth parameters of the newly isolated *S. cerevisiae* ETP53, *P. fermentans* ETP22, and *K. marxianus* ETP87 using RSM and conventional methods for efficient ethanol production.

## 2 Materials and methods

### 2.1 Sampling

**Fig. 1:**
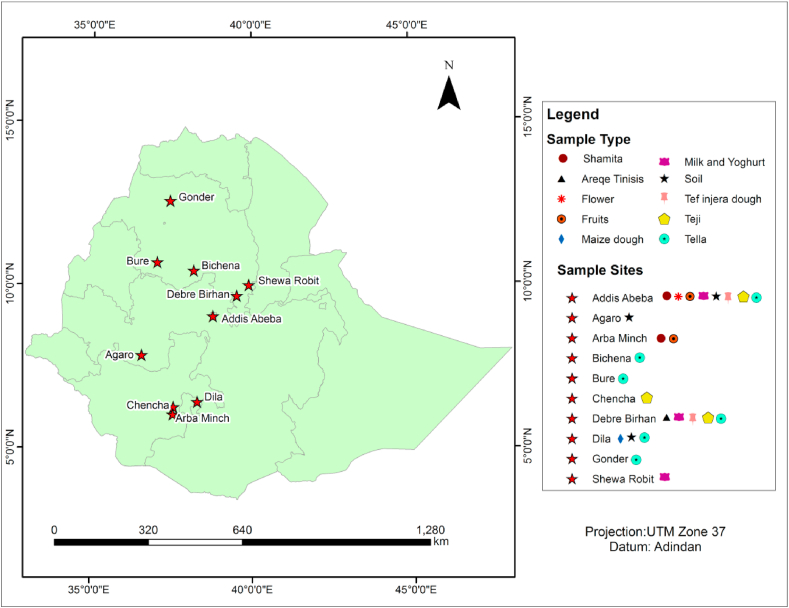
Sampling sites for yeast isolation.

Samples were collected from traditional foods and beverages such as *tella*, *teji*, *shamiata, areqe tinisis,* teff *enjera* dough, maize dough, milk and ergo, soil, fruits, compost and nectar of different flowers from different sampling sites in Addis Ababa, Debre Berhan, Dilla, Bure, Bichena, Gonder, Chencha-Dorze, Shoa Robit, Agaro, Dilla and Sebeta in Ethiopia (Fig. 1).

### 2.2 Isolation

The yeasts were isolated on YPDA medium (Yeast extract, 10; peptone, 20; dextrose, 20; and agar, 20 g/L) containing chloramphenicol (0.01 g/L) to inhibit bacterial growth [20]. To isolate five carbon-utilizing yeasts, dextrose was substituted by ribose (20 g/L). Aliquots (100 μL) of samples from processed samples were prepared at appropriate dilutions and 100 μL of the suspensions were spread on YPDA agar media and incubated at 30 °C for 3 days [20].

### 2.3 Screening of ethanol producing yeasts

The isolates were screened for ethanol production using standard methods [21]. For screening of 5-carbon utilizing yeasts, dextrose was replaced by ribose as before. 0.5 mL of active yeast cells (approximately 10^6^ cells) for each isolate were inoculated into the media in Durham test tubes. They were incubated at 30 °C for 10 days without agitation. The fermenting yeasts were further screened based on time that completely displace 1 mL Durham tube with gas in 12, 24, 48 and 72 h. They were, then, screened based on ethanol concentration that they produced [21]. Finally, they were compared with instant baker's yeast.

### 2.4 Growth and fermentation at different carbon sources

The effects of different carbon sources (xylose, arabinose, raffinose, trehalose, mannitol, cellobiose, galactose, fructose, maltose, lactose, mannose, sucrose, and starch (each 2% w/v) on ethanol production in 100 mL media containing 1 g yeast extract and 2 g peptone were investigated [23]. During solid media preparation, ethanol was added after sterilization before pouring and mixed very well. Solid media were used for growth evaluation using colony diameter whereas the fermentation was tested using inverted 1 mL Durham test tubes containing the same media.

### 2.5 Identification of yeast species

#### 2.5.1 DNA extraction

Isolates ETP53, ETP37, ETP87, ETP94, ETP50, ETP89, ETP22, and ETP122 that showed relatively good ethanol production were selected and identified using standard genetic methods. Accordingly, DNA was extracted by sub-culturing yeast cells on YM agar (yeast extract, 3; malt extract, 3; dextrose, 10, peptone, 5; and agar 20 g in 1000 mL deionized water), plate at 20 °C for 5–7 days; then 50 μL volume of cell mass was harvested in a microtube suspended in 200 μL lysis solution (1% [w/v] Yatalase™ (TAKARA Bio Inc.), 1% [v/v] RNase A solution (Qiagen), 10 mM potassium phosphate, 10 mM EDTA, 0.8 M NaCl, pH 7.0), and incubated at 37 °C for 1.5 h. Approximately, 50 μL of Φ 0.8 mm glass beads and 67 μL of SDS/ProK solution (8% [w/v] SDS, 300 U of Proteinase K (Nacalai Tesque), 5 mM Tris-HCl, 0.5 mM EDTA, 50 mM NaCl, pH 8.0) were added to each tube. The tube was vortex-mixed for 1.5 min, and incubated at 60 °C for 10 min. After this, 87 μl of 3 M sodium acetate (pH 5.2) solution was added, vortex-mixed, and chilled on ice.

The tube was centrifuged at 15,000 rpm for 5 min at 4 °C from which 70 μL of the supernatant was transferred to a well of AcroPrep™ 96 Multi-Well Filter Plate with 3.0 μM glass fiber media/0.2 μM Bio-Inert® membrane, natural housing (PALL Life Science), to which 110 μL of isopropanol was added and mixed well by pipetting. After incubation for 3 min at room temperature (15–20 °C), the filter plate was vacuumed with a vacuum manifold device. The well was rinsed with 200 μL of 70% [w/v] ethanol twice in vacuum. After the filter plate was air-dried, 60 μL of TE buffer (10 mM Tris-HCl, 1 mM EDTA, pH 8.0) was poured in the well, and incubated for 3 min. The filter plate was placed onto a new 96-well plastic plate. Two plates were centrifuged at 3000 rpm for 5 min at room temperature. The DNA was resuspended with TE buffer, centrifuged twice, and kept at −20 °C for further use.

#### 2.5.2 Sequencing of LSU rRNA

The DNA was sequenced using NL1 (5′-GCATATCAATAAGCGGAGGAAAAG-3′) and NL4 (5′-GGTCCGTGTTTCAAGACGG-3′) as PCR primers for amplification of D1/D2 region of LSU rDNA [24]. PCR amplification was performed in 20 μL reaction, containing 10 μL of GoTaq® Green Master Mix (Promega), 10 pmol of each primer, and 2 μL of 1–20 ng/μL extracted DNA, on GeneAmp® PCR System 9700 (Applied Biosystems) or iCycler (BioLad). The PCR program was as follows; an initial denaturation at 94 °C for 5 min, followed by 36 cycles of 30 s at 94 °C, 30 s at 52 °C, 1 min at 72 °C, and a final extension of 5 min at 72 °C. The PCR-amplified fragment was visualized by electrophoresis on agarose and staining with ethidium bromide. Purification of the fragment was performed using MinElute® 96 UF PCR Purification Kit (Qiagen) according to the manufacturer's instructions. The purified fragment was resuspended in 50 μL of 10x diluted TE buffer. The nucleotide sequences of PCR-amplified fragment were determined by Sanger-sequencing using the ABI PRISM® 3130*xl* Genetic Analyzer (Applied Biosystems) following the manufacturer's instruction. Sequence data were corrected by manual inspection whenever needed, and aligned using BioEdit Sequence Alignment Editor version 7.1.3.0 [26].

#### 2.5.3 Identification of yeast isolates

The partial sequence (D1/D2 region) was edited by BioEdit as explained above. Genetic identification of yeast isolates were done by blasting isolates’ D1/D2 sequences against GenBanks such as National Center for Biotechnology Institute (NCBI) (https://blast.ncbi.nlm.nih.gov/Blast.cgi) and CBS database, Westerdijk Institute (http://www.westerdijkinstitute.nl/). The percentage of similarity between partial sequence result of the yeast isolates was compared with sequences similar to isolates accessed from GenBanks (NCBI and CBS) and created by free Mega6 and BioEdit software [3,24].

### 2.6 Yeasts sedimentation rate

One mL of 24 h culture grown on YPD was transferred to 1.5 mL Eppendorf tubes and centrifuged at 14000× g (Eppendorf centrifuge 5418 R, Germany) for 10 min and the pellets were resuspended in 1 mL NaCl (0.89%) solution for 2 h. The optical density was measured at 600 nm using Jenway 6405 UV/Vis Spectrophotometer (United Kingdom). The sedimentation rate was expressed according to Ref. [27].

Equation 1 $$\text{\% of sedimentation}=\left(1-\frac{\text{total drop in OD reading after }2\text{ hour}}{\text{OD reading at }0\text{ hour }}\right)\times 100\text{\%}$$

2.7Measurement of ethanol tolerance

Measurement of cell viability after ethanol shock treatment was employed to evaluate ethanol tolerance [28]. Accordingly, overnight yeast cultures on YPD were harvested and washed two times with de-ionized water. After centrifugation at 13,000 QUOTE × ×g (Eppendorf centrifuge 5418 R, Germany) the yeast pellets were diluted with acetate buffer (pH 5.0) and exposed to 10, 15 and 20% (v/v) ethanol in the same acetate buffer. They were incubated at 30 °C in water bath (Clifton, England) at 150 forth and back shakings per minute for 2 h. The samples were serially diluted to 10^−5^ using acetate buffer (pH 5.0) from which 100 μL portion of diluent were spread to YPD agar plate and incubated at 30 °C for 4 days to count colonies. The percentage of colonies was taken as a measure for ethanol tolerance [28].

Equation 2 $$\text{Survived}\;\text{percentage}=\frac{\text{Number of survived cells after exposure}}{\text{Number of unstressed }\left(\text{unexposed}\right)\text{cells control}}\times 100\text{\%}$$

2.8Optimizing growth variables

Experiments to analyze the effects of temperature (30, 35, and 40 °C) and pH (4, 5, and 6) on ethanol production were run using YPD under batch fermentation for 48 h [29]. Samples were taken at 5, 20, 27 and 48 h for ethanol analysis for yeasts inoculated in YPD containing 2% dextrose. On other experimental runs, 24, 48 and 72 h were considered as sampling times for yeasts grown in YPD containing 4% dextrose.

The response surface methodology experiments were undertaken by cultivating the yeasts in 250 mL Erlenmeyer flask containing 100 mL YPD by applying the Central Composite Design (CCD) using Design Expert 7.0.0 (Stat Ease, Inc., Minneapolis, USA). The number of experiments generated by CCD was 20 from 3 factors at 3 levels with six replications at center point to evaluate the pure error. The performance of the system was assessed by the response (ethanol produced in g/L). The optimization process employed both quadratic and linear model [18]; the model was given as.

Equation 3 $$\text{Y}=\beta_{0}+\beta_{1}{\text{X}}_{1}+\beta_{2}{\text{X}}_{2}+\beta_{3}{\text{X}}_{3}+\beta_{1}\beta_{2}{\text{X}}_{1}{\text{X}}_{2}+\beta_{1}\beta_{3}{\text{X}}_{1}{\text{X}}_{3}+\beta_{2}\beta_{3}{\text{X}}_{2}{\text{X}}_{3}+\beta_{11}{\text{X}}_{1}^{2}+\beta_{22}{\text{X}}_{2}^{2}+\beta_{33}{\text{X}}_{3}^{2}+\varepsilon$$

Where Y = ethanol produced in g/L (dependent output); β_0_ = intercept (the constant process effect in total); β_1_, β_2_, and β_3_ = Linear, quadratic and interaction regression coefficients for temperature, pH and time, respectively (parameters); X_1_, X_2_, and X_3_ = independent variable for temperature (degree centigrade), pH, and time (hours), respectively; and *ε* = random experimental error assumed to have a zero mean.

The analysis of variance (ANOVA) and the significance of the model equation were determined by the coefficient of determination ($R^{2}$), *p*-value and *F*-test using Design Expert. The response surface was optimized for the maximum ethanol production [29].

### 2.9 Analytical methods

The reducing sugars were estimated by DNS method [30]. In order to determine the ethanol, the fermentation samples were centrifuged at 14000× g for 5 min. The supernatant was analyzed by Gas Chromatography (Agilent 6890 N) coupled with a mass spectrometer (MS) with headspace autosampler (Agilent 7694E). The GC-MS was equipped with Mass Selective Detector (Agilent 5973 Network) and a polar polyethylene glycol (PEG) phase DB-wax122-7032 capillary column. Helium was used as a carrier gas. The flow rate for the column was 1 mL/min. The column temperature was held at 40 °C for 4 min, and then raised to 240 °C at 40 °C/min; the inlet temperature was 220 °C.The GC was operated with 20:1 split injection of the headspace.

The mass-to-charge ratios (m/z) for ethanol was 31–45 m/z range with the retention time of 3.36 min and for furfural 32–95 m/z range with the retention time of 7.64 min. The MS Quadrupole, MS source and transfer line temperature were 150 °C, 230 °C, and 250 °C, respectively. The conditions of the headspace autosampler were 25min for the GC cycle time, 10 min for the vial equilibration time, 0.5min for the pressurization time, 1min for the injection time and a constant vial pressure of 14.0 psi. The temperatures were set at 110 °C for the transfer line to the column and at 90 °C for the loop. The equilibration temperature was 80 °C for 10 min. Fermentation samples (200 μL) were put in 10 mL headspace vials.

## 3 Results and discussion

### 3.1 Yeast isolation

A total of 211 yeast colonies were collected from traditional alcoholic beverages, *enjera* and maize dough, *ergo* and milk, soil and compost, flowers and fruits. The highest yeast counts were recorded from beverages *tella*, *teji*, and fermented enjera-maize dough with population of 4.6$\times 10^{5}$, 2.4$\times 10^{5}$, and 2.7$\times 10^{5}$ CFU, followed by population density of 9.9$\times 10^{4}$, $\;6.6\times 10^{4},\;1.4\times 10^{4}$ , 1.2$\times 10^{4}$, and 1.1$\times 10^{4}$ yeast cells from *areki tensis*, *shamita*, flowers, milk and *ergo*, and compost, respectively (Table 1)*.* .

**Table 1:** Yeasts density and number of dextrose fermenters.

Samples	CFU	Number of colony types	Number of dextrose fermenters	Percentage of dextrose fermenters
*Tella*	4.6$\times 10^{5}$^b^	34	21	62
*Teji*	2.4$\times 10^{5}$^b^	24	19	79
*Araki tensis*	9.9$\times 10^{4}$^b^	19	16	84
Milk and *ergo*	1.2$\times 10^{4}$^b^	13	6	46
Enjera and maize dough	2.7$\times 10^{5}$^b^	11	9	82
*Shamita*	6.6$\times 10^{4}$^b^	8	6	75
Fruits	8.9$\times 10^{3}$^a^	30	10	33
Flowers	1.4$\times 10^{4}$^a^	24	9	38
Soil	9.8$\times 10^{3}$^a^	33	22	67
Compost	1.1$\times 10^{4}$^a^	15	8	53
			126	60

a CFU per gram whereas.

b CFU per milliliter.

The number of the different types of yeasts (diversity) was in the range of 8 colony types from compost to that of 34 colony types obtained from tella (Table 1). Thus, the number of colony types can be categorized into low diversity (8–20 different colony types) recorded from *shamita, areqe tinisis,* Milk and *ergo,* compost, and *enjera* and maize dough, and medium (20–30 colony types) recorded from samples of Teji, flowers, and fruits and high diversity colony types (>30) from tella and soil (Table 1).

Accordingly, the number of isolates (34 colonies) detected from tella was similar to the number of isolates detected from soil (33 isolates) and fruits (30 isolates) (Table 1). Similarly, the higher population density exhibited from maize dough showed less diversity (11 colonies). Although the number of yeast colonies was higher from *tella*, *shamita*, *teji*, flowers and maize dough, and lower in fruits and soil than other sources, they did not match the number of colony types (diversity) indicating that population density did not necessarily corroborate with species diversity.

### 3.2 Yeast screening by dextrose fermentation

Among 211 yeasts isolated from all samples, 126 yeasts (60%) were able to produce gas from dextrose (Table 1) which was the confirmatory test for ethanol production by yeasts**.** The existence of high dextrose fermentative yeasts (60–80%) were recorded from *tella*, *teji*, *areqe tinisis* and *shamita, enjera* and maize dough, and soil; whereas 30–50% ethanol producing isolates were recorded from other samples. It was interesting to note that although fruits and flowers contained diverse groups of yeasts, the ethanol producers were relatively lower (<40%) than the other yeasts isolated from beverages (Table 1). On the contrary, most of the yeast fermenters were detected (60–80%) from fermented food and beverages indicating that the ethanol fermenters were dominated by *S. cerevisiae* [31] and that is why most of the yeasts (>50) isolated from fermented products are fermentation positive. This suggests that the probability of getting good ethanol-producing yeast is higher in these samples [32].

### 3.3 Molecular identification of yeast isolates

The yeasts identity is commonly identified by highly similar gene sequences of the 5′ region of the large subunit (LSU); the D1/D2 domain is the name given for a region around 600 nucleotides at the 5’ end of a large subunit of (26 S) rDNA. Furthermore, D1/D2 region is highly conserved domain of LSU; therefore, the region is intensively utilized for rapid and accurate species identifications [3,24]. [3] also noticed that most of the yeast partial sequences in GenBanks are D1/D2 domain and this further urges to use D1/D2 regions for yeast identification.

D1/D2 domain of 26 S rRNA of ETP37, ETP50, ETP53, ETP89, and ETP94 was 100% similar with *S. cerevisiae* NS G-48, *S. cerevisiae* NC007, *S. cerevisiae* CBS 2984, and *S. cerevisiae* CBS 5493 from NCBI data base; they were also similar with *S. cerevisiae* CBS 6308 (CBS data base) with 100% confidentiality. Based on sequence analysis of the first 600 nucleotides from 5’ end of LSU of rRNA and comparison from NBCI blast, ETP87 was found to be 100% alike with *K. marxianus* U-MF11, *K. marxianus* Y12, *K. marxianus* CBS 5672 and *K. marxianus* IMAU6Y146 (DX9-2). D1/D2 region of ETP22 matched 100% with *P. fermentans* A5, *P. fermentans* KDLYH2-3, and *P. fermentans* CBS 5662 (from NCBI). The overlapping between ETP22 and *P. fermentans* CBS 6662 and CBS 5663 (CBS database) was 100 and 99.825% respectively. The same region sequence analysis of ETP122 exhibited that the sequence was 100% identical with *C. humilis* H17 and *C. humilis* IMAU Y10085 that were accessed from NCBI. The resemblance of ETP122 and *C. milleri* CBS 6897 from CBS data base was 99.825%.

### 3.4 Ethanol production by local isolates and commercial baker yeast

The different isolates were screened for efficient ethanol production, of which 8 isolates were selected and characterized (Table 2). These yeast isolates were categorized into four genera *Saccharomyces cerevisiae* containing four isolates; ETP37, ETP50, ETP53, ETP89 isolated from beverages tella, teji, and shamita and ETP94 isolated from flower sample. The others were identified as *Kluyveromyces marxianus,* ETP87 isolated from the milk product, ergo, *Pichia fermentans,* ETP22 isolated from compost, and *Candida humilis* (*milleri*), ETP122 isolated from enjera dough. These isolates produced ethanol ranging from 5.37 g/L (ETP122) to 9.0 g/L (ETP53) **(**Table 2**)**. Thus, the *Saccharomyces cerevisiae* isolates were dominant both in terms of number (65%) and production of alcohol (7.67g/L-9.0 g/L). It is interesting to note that the non-*Saccharomyces* yeast, *K. marxianus* was as equally efficient in alcohol production (7.97gL) as that of *S. cerevisiae* isolates.

**Table 2:** Ethanol from 2% dextrose (w/v) by yeasts isolated from different sources.

Yeast isolates	Isolated from	Molecularly identified as	Ethanol produced (g/L)	
Baker yeast	Commercial instant dry yeast	*Saccharomyces cerevisiae*	8.43 ± 0.41	
ETP22	Compost	*Pichia fermentans*	6.43 ± 0.31	
ETP37	Teji	*Saccharomyces cerevisiae*	8.10 ± 0.28	
ETP50	Teji	*Saccharomyces cerevisiae*	7.67 ± 0.25	
ETP53	Tella	*Saccharomyces cerevisiae*	9.00 ± 0.17	
ETP87	Ergo	*Kluyveromyces marxianus*	7.97 ± 0.30	
ETP89	Shamita	*Saccharomyces cerevisiae*	7.60 ± 0.33	
ETP94	Flower	*Saccharomyces cerevisiae*	7.80 ± 0.40	
ETP122	Enjera Dough	*Candida humilis* (*milleri*)	5.37 ± 0.11	
	

### 3.5 Growth and fermentation of different carbon sources by the selected yeasts isolates

Almost all of the selected isolates were capable of growing and vigorously fermenting glucose, fructose and galactose, and majority of them utilized mannose, raffinose, maltose and sucrose (Table 3). One or two isolates weakly grew on one of xylose, arabinose, trehalose, mannitol, and starch with or mild fermentation.

**Table 3:** Growth and fermentation of yeast isolates in different sugar sources.

Sugar source	Yeast Isolates															
	*P*. *fermentans* ETP22		*S. cerevisiae* ETP37		*S. cerevisiae* ETP50		*S. cerevisiae* ETP53		*K. marxianus* ETP87		*S. cerevisiae* ETP89		*S. cerevisiae* ETP94		*C. humilis* ETP122	
	Growth	Fermentation	Growth	Fermentation	Growth	Fermentation	Growth	Fermentation	Growth	Fermentation	Growth	Fermentation	Growth	Fermentation	Growth	Fermentation
Dextrose	+	+++	+	+++	+	+++	+	+++	+	+++	+	+++	+	+++	+	++
Galactose	+	–	+	++	+	++	+	+++	+	++	+	+++	+	+++	+	+
Fructose	+	++	+	++	+	+++	+	+++	+	+++	+	+++	+	+++	+	+
Mannose	+	+++	+	++	+	++	+	++	–	+++	+	+++	+	+++	–	–
Xylose	+	–	–	–	–	–	–	–	+	–	–	–	–	–	_	–
Arabinose	–	–	–	–	–	–	–	–	+	–	–	–	–	–	–	–
Trehalose	–	–	W	D	W	D	W	D	W	–	–	–	–	–	+	D
Raffinose	+	–	+	++	+	++	+	++	+	++	+	–	+	–	+	++
Cellobiose	+	–	–	–	+	D	–	–	+	–	–	–	W	–	+	–
Mannitol	–	–	W	–	W	–	W	–	W	D	W	D	–	–	+	+
Maltose	+	–	+	+++	+	+++	+	+++	+	+	+	+++	+	D	+	–
Lactose	–	–	–	–	–	–	–	–	+	+++	–	–	–	–	+	–
Sucrose	–	–	+	++	+	++	+	++	+	+++	+	+++	+	–	–	_
Starch	–	–	W	–	W	–	W	–	–	–	W	–	W	–	+	D
Ethanol	+	–	+	–	+	–	+	–	+	–	+	–	+	–	–	–
**Fermentation**: +++ fermentation less than 24 h; ++, fermentation within 24–48 h; +, 48 h to 7 days; hrs; D, Delayed (>7 days) positive response; -no fermentation **Growth**: + positive response; W, weak positive response; - negative response (no growth)																

Out of the isolates, *K. marxianus* ETP87 was capable of utilizing the maximum number of sugars (78%) and vigorously fermented glucose, galactose, fructose, and raffinose, lactose and sucrose unlike the other isolates that were limited to grow on and ferment fewer substrates. The different *S. cerevisiae* strains were consistent in their growth and fermentation of the majority of the sugars (50%–60%).

*K. marxianus* ETP87 was the only yeast that ferments lactose besides glucose, galactose, fructose, maltose and sucrose within 24 h. It could be good candidate for ethanol production from whey. *K. marxianus* would only ferment dextrose and assimilate dextrose and xylose among carbon sources tested [21]. [23] also reported that *K. marxianus* had a capability to grow on and ferment glucose, galactose, maltose, sucrose, lactose, trehalose and raffinose despite variation among different strains.

The other non-Saccharomyces yeast *P. fermentans* ETP22 and *Candida humilis* (*milleri*) ETP122 were capable of growing on the majority of the sugar substrates (60–65%), but fermented fewer (20–30%) of the substrates indicting that they were not efficient alcohol fermenters. On the other hand, however, *P. fermentans* ETP22 which was isolated from compost, fermented glucose, mannose and fructose though dextrose fermentation was rarely reported before [21,23]. This isolate was able to grow on xylose even if it didn't ferment it and can be good candidate to produce biomass from acid hydrolysate of lignocellulose since five carbon sugars are dominant in acid hydrolysates of such substrates. It also grew better on starch than others and it might be due to long time adaptation since it was isolated from *enjera* dough. Similarly, the dominant yeast in the dough was found to be *C. humilis* [33]. All the isolates except *C. humilis* ETP122 were grown on ethanol and hence they might decrease the ethanol titer produced when the sugar in the growth media is depleted [34]. studied alcohol dehydrogenases (Adh) in yeasts and confirmed that yeast later consumes the accumulated ethanol, exploiting Adh2, an Adh1 homolog differing by 24 (out of 348) amino acids.

### 3.6 Yeast flocculation and sedimentation

The sedimentation rate of *S. cerevisiae* ETP50, *S. cerevisiae* ETP89, and *S. cerevisiae* ETP94 was 81% whereas *S. cerevisiae* ETP53, *S. cerevisiae* ETP37, *P. fermentans* ETP22, *K. marxianus* ETP87, and *C. humilis* ETP122 were 83%, 79%, 78%, 74%, and 6.8%, respectively. The average sedimentation rate of the yeast species was 80% under natural conditions, except C*. humilis* ETP122. This was much better than the sedimentation rate of 70% recorded in wine yeast by applying external flocculating agent such as sucrose and sorbitol to the growing media [27]. This difference might be due to the inability to express *FLO* genes in *C. humilis* ETP122 since *FLO* gene is present among industrial yeast strains though its expression varies among strains [36]. Generally, all the 7 yeast isolates can be good in terms of cell separation for industrial production of yeast biomass and alcohol.

Cations (Ca^2+^, Rb^+^, Cs^+^, Fe^2+^, Co^2+^,Cu^2+^, Ni^2+^, Zn^2+^, Cd^2+^, Al^3+^, Mg^2+^ and Mn^2+^), lower pH (3–5), moderate aeration, agitation, and high cells load (>4 × 10^7^ cells per mL) induce and promote yeast flocculation; however, higher pH, higher temperature, fermentable sugars, EDTA and high ethanol facilitate loss of flocculation [48].

### 3.7 Ethanol tolerance

**Fig. 2:**
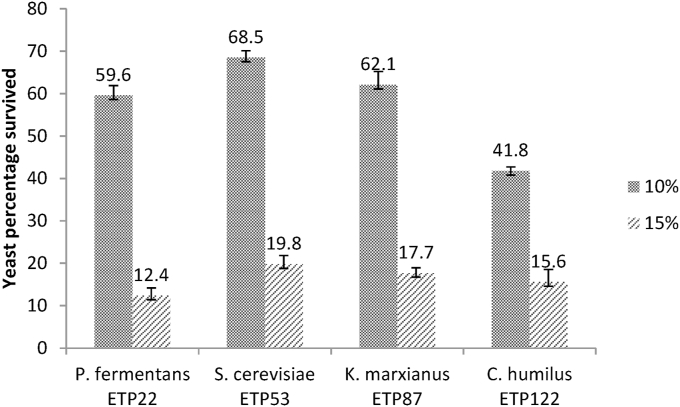
Ethanol tolerance test for 10, and 15% (v/v) ethanol concentration.

Based on the higher ethanol they produced and by considering diversity, four selected yeast strains from each genus were evaluated for their ethanol tolerance (shock treatment) on YPD medium containing 10, 15, and 20% extraneous ethanol (Fig. 2). The highest cell viability of 68% was recorded from S*. cerevisiae* ETP53 at 10% ethanol concentration, followed by *K. marxianus* ETP87, *P. fermentans* ETP22 and *C. humilis* ETP122 strains with survival rates of 65%, 60%, and 40%, respectively. However, the viability of the strains decreased with the same pattern at 15% ethanol concentration within the range of 10–20%. Most yeast died at 20% ethanol concentration. Similarly [37], showed most of *S. cerevisiae* strains died at 12.5% ethanol after 3 h of incubation. The higher ethanol concentration affects hydrophobic proteins present in cell, vacuolar, lysosomal, mitochondrial, nuclear membranes and endoplasmic reticulum besides hydrophilic proteins in cytoplasm and nucleoplasm and hence it influences the integrity of membranes and their functions [12].

This study showed that non-*Saccharomyces* yeasts, *K. marxianus* ETP87 and *C. humilis* ETP122 showed similar pattern of tolerance with *S. cerevisiae* strain to 10% ethanol which was higher than their tolerance to 6% reported by Ref. [38]. [16] also showed that non-*Saccharomyces K. marxianus* tolerated relatively lower (5–7% v/v) ethanol concentration than *S. cerevisiae* (8–10%). However, other studies also indicated that non-*Saccharomyces* species had similar (or even higher) ethanol tolerance than *S. cerevisiae* and a stronger resistance to fermentation conditions than *S. cerevisiae* [17,20].

Even though viable yeast cells were found in the medium containing 15% exogenous ethanol in this study [39], concluded that yeast viability at increased ethanol concentrations may not necessarily lead to the ability to produce ethanol at these conditions. This should be substantiated by additional studies on different substrates and at different incubation time, and concentration of ethanol to use non-*Saccharomyces* yeasts for large scale production of ethanol. However, De la Torre-González et al. [17]) reported that the non-*Saccharomyces* yeasts are unable to grow at YPD containing more than 10% (v/v) ethanol.

### 3.8 Ethanol productivity, yield and efficiency

In this study, *S. cerevisiae* ETP53 (92%) and *K. marxianus* ETP87 (84%) were more efficient for ethanol production from dextrose than *P. fermentans* ETP22 (62%) within 5–20 h at 2% (w/v) glucose. The highest ethanol productivity was observed in *S. cerevisiae* ETP53 (1.38 g/L/hr) within 5 h which was 14 times higher than *P. fermentans* ETP53 and *C. humilis* ETP122 within the same incubation time. Thus, only *S. cerevisiae* ETP53 produced economical ethanol within 5 h with 84% efficiency (Table 4). Therefore, ethanol production (concentration), productivity, and yield cannot be used interchangeably to describe ethanol quantification.

**Table 4:** Ethanol productivity, yield and efficiency of the three isolates grown in YPD at 2% glucose.

Isolate	Time (hr)	Ethanol conc. g/L	Productivity (g/L/hr)	Yield (g/g)	Efficiency (%)
*P. fermentans* ETP22	5	0.52	0.1	0.03	0.06
	20	1.13	0.06	0.07	13.73
	27	5.15	0.19	0.32	62.75
	48	4.19	0.09	0.26	50.98
*S. cerevisiae* ETP53	5	6.9	1.38	0.43	84.13
	20	7.5	0.38	0.47	92.16
	27	5.88	0.22	0.37	72.55
	48	5.9	0.12	0.37	72.55
*K. marxianus* ETP87	5	0.67	0.13	0.04	0.08
	20	6.88	0.34	0.43	84.13
	27	6.95	0.26	0.43	84.13
	48	3.44	0.07	0.22	43.14
Theoretical				0.51	100

### 3.9 Response surface analysis for temperature, pH and time optimization

Based upon prior results from other studies [18,29] and data from samples for isolation, temperature (30–40 °C), pH (4.0–6.0), and incubation time (24–72 h) ethanol optimization was undertaken using RSM.

#### 3.9.1 *S. cerevisiae* ETP53

The actual yield and predicted value generated by the model is given in Table 5. The correlation between actual and predicted value for *S. cerevisiae* ETP53 was 0.9846. Therefore, the deviation between the actual and predicted value was low.

**Table 5:** Central composite design matrix for three independent variables with actual and predicted values of ethanol produced from 40 g/L dextrose by *S. cerevisiae* ETP53.

Standard order	Run order	Temperature (^o^C)	pH	Time (hours)	Actual value (g/L)	Predicted value (g/L)	Residual
1	8	30	4	24	10.1	8.95	1.15
2	3	40	4	24	1.4	1.34	0.06
3	11	30	6	24	9.2	8.97	0.23
4	5	40	6	24	1.5	2.21	−0.71
5	18	30	4	72	19.4	17.14	2.26
6	15	40	4	72	2.3	0.98	1.32
7	19	30	6	72	18.9	17.42	1.48
8	6	40	6	72	2.5	2.11	0.39
9	9	26.59	5	48	17.36	19.66	−2.30
10	17	43.41	5	48	0.5	0.38	0.12
11	4	35	3.32	48	3.2	5.30	−2.10
12	2	35	6.68	48	6.18	6.26	−0.08
13	12	35	5	7.64	4.8	4.48	0.32
14	14	35	5	88.36	8.79	11.29	−2.50
15	16	35	5	48	16.46	16.19	0.27
16	20	35	5	48	15.99	16.19	−0.20
17	13	35	5	48	16.22	16.19	0.03
18	7	35	5	48	16	16.19	−0.19
19	10	35	5	48	16.32	16.19	0.13
20	1	35	5	48	16.54	16.19	0.35

Table 6 shows that the quadratic model employed here was fit (*p* < 0.00001). The degree of significance showed that temperature had greatest effect whereas pH was the lowest. However, pH played significant role in the interactions even if it was the lowest compared to temperature and time.

**Table 6:** ANOVA for response surface (temperature, pH and time) quadratic model of *S. cerevisiae* ETP53.

Source	Sum of Squares	df	Mean Square	F Value	p-value Prob > F
Model	870.71	9	96.75	35.35	<0.0001
A-Temperature	448.41	1	448.41	163.86	<0.0001
B-pH	1.12	1	1.12	0.41	0.5366
C-Time	55.82	1	55.82	20.40	0.0011
AB	0.36	1	0.36	0.13	0.7239
AC	36.55	1	36.55	13.36	0.0044
BC	0.03	1	0.03	0.01	0.9170
A^2^	68.64	1	68.64	25.08	0.0005
B^2^	195.31	1	195.31	71.37	<0.0001
C^2^	124.33	1	124.33	45.43	<0.0001
Residual	27.37	10	2.74		
Lack of Fit	27.10	5	5.42	102.68	<0.0001
Pure Error	0.26	5	0.05		
Cor Total	898.07	19			

Though the model was sufficient (*p* < 0.0001) to explain the interactions, not all effects of interactions were significant (*p* < 0.05) for ethanol production (Table 6); nevertheless, all were included in the model equation because adding insignificant value to a number will not change it significantly. The second order polynomial equation to produce ethanol (Y) as a function of temperature (X_1_), pH (X_2_) and time (X_3_) was obtained as

Equation 4 $$\text{Y}=-181.73+5.61{\text{X}}_{1}+35.49{\text{X}}_{2}+1.18{\text{X}}_{3}+0.04{\text{X}}_{1}{\text{X}}_{2}-0.02{\text{X}}_{1}{\text{X}}_{3}+2.6{\text{X}}_{2}{\text{X}}_{3}-0.09{\text{X}}_{1}^{2}-3.68{\text{X}}_{2}^{2}-5.1{\text{X}}_{3}^{2}$$

Interactions among temperature, pH and time.

**Fig. 3:**
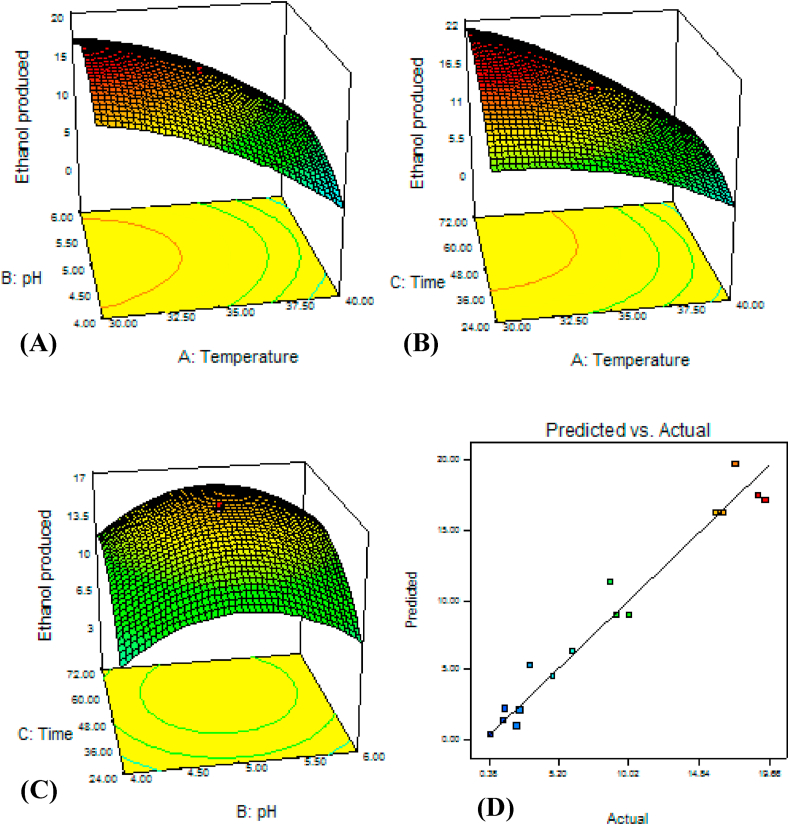
*S. cerevisiae* ETP53's response surface and contour plate of temperature vs. pH (A), temperature vs. time (B), pH vs. time (C), and correlation plot showing the distribution of actual (observed) and predicted values of ethanol (g/L) produced from 40 g/L dextrose.

Fig. 3 shows the response surface curve with contour plots for optimization of ethanol production as a function of temperature, pH and time. Maximum ethanol was produced at the mild pH (5.5) and lower temperature (less than 33 °C) (Fig. 3 A). Similarly [18], reported that optimum ethanol production using *S. cerevisiae* was obtained at pH 5.5. Most favorable pH of *S. cerevisiae* for ethanol production is ranged from 4.0 to 5.0 [40].

The increase in temperature above 35 °C significantly reduced the ethanol yield (Fig. 3 B). [16] showed that the fermentation efficiency of *S. cerevisiae* at a temperature more than 35 °C was significantly low. The data also showed that higher incubation time (greater than 38 h) and lower temperature (less than 33 °C) resulted in optimal yield (Fig. 3 B). Similarly [40], reported that ethanol concentration was found peak (18.3 g/L) at 30 °C within 48 h, but higher temperature reduced both ethanol production as well as biomass. The temperature range between 30 and 35 °C was best for ethanol production by *S. cerevisiae* [41]. In pH and time interaction, best ethanol was produced nearly (a little higher time) at the center (Fig. 3C) and then the yield decreased at any direction in the model. [40] investigated the fermentation time effect on ethanol production and they found that best yield was attained at 48–72 h. The maximum ethanol (20.93 g/L) was attained at temperature (30.1 °C), pH (5.13), and incubation time (58.97 h) (Fig. 3).

#### 3.9.2 2*. K. marxianus* ETP87

A 3-level 3-factors central composite design (CCD) was performed with different combinations of temperature, pH and time to upgrade ethanol production by the yeast. Table 7 shows the strong correlation value between observed and predicted ethanol production generated by the model.

**Table 7:** Central composite design matrix for three independent variable with actual and predicted values of ethanol produced from 40 g/L dextrose by *K. marxianus* ETP87.

Standard order	Run order	Temperature (^o^C)	pH	Time (hours)	Actual value (g/L)	Predicted value (g/L)	Residual
1	13	30	4	24	7.85	6.60	1.25
2	11	40	4	24	9.3	8.65	0.65
3	2	30	6	24	7.65	6.84	0.81
4	15	40	6	24	8.56	7.38	1.18
5	1	30	4	72	16.98	16.10	0.88
6	12	40	4	72	16.05	14.80	1.25
7	6	30	6	72	13.62	12.22	1.40
8	5	40	6	72	10.22	9.41	0.81
9	10	26.59	5	48	9.84	11.43	−1.59
10	19	43.41	5	48	9.47	10.79	−1.32
11	16	35	3.32	48	11.13	12.53	−1.40
12	17	35	6.68	48	6.7	8.20	−1.50
13	14	35	5	7.64	4	5.32	−1.32
14	9	35	5	88.36	13.43	15.01	−1.58
15	4	35	5	48	15.78	15.38	0.40
16	20	35	5	48	15.52	15.38	0.14
17	3	35	5	48	15.63	15.38	0.25
18	18	35	5	48	15.11	15.38	−0.27
19	7	35	5	48	15.83	15.38	0.45
20	8	35	5	48	14.9	15.38	−0.48

Unlike *P. fermentans* ETP22 and *S. cerevisiae* ETP53, time and temperature had the highest and lowest impact on ethanol production in *K. marxianus* ETP87, respectively. The regression analysis of the model (Table 8) showed that 92.02% of the variation could be explained. The analysis of ANOVA and multiple regression resulted second order polynomial equation. In the equation, Y (ethanol produced) was the function of temperature (X_1_), pH (X_2_), and time (X_3_) and it is given as

Equation 5 $$\text{Y}=-143.51+4.9{\text{X}}_{1}+21.13{\text{X}}_{2}+0.89{\text{X}}_{3}-0.08{\text{X}}_{1}{\text{X}}_{2}-6.97{\text{X}}_{1}{\text{X}}_{3}-0.04{\text{X}}_{2}{\text{X}}_{3}-0.06{\text{X}}_{1}^{2}-1.77{\text{X}}_{2}^{2}-3.2{\text{X}}_{3}^{2}$$

**Table 8:** ANOVA for response surface (temperature, pH and time) quadratic model of *K. marxianus* ETP87.

Source	Sum of Squares	df	Mean Square	F Value	p-value F
Model	258.14	9	28.68	12.81	0.0002
A-Temperature	0.49	1	0.49	0.22	0.6493
B-pH	22.63	1	22.63	10.10	0.0098
C-Time	113.49	1	113.49	50.67	<0.0001
AB	1.13	1	1.13	0.51	0.4933
AC	5.59	1	5.59	2.50	0.1451
BC	8.51	1	8.51	3.80	0.0799
A^2^	32.89	1	32.89	14.68	0.0033
B^2^	45.27	1	45.27	20.21	0.0012
C^2^	48.95	1	48.95	21.85	0.0009
Residual	22.40	10	2.24		
Lack of Fit	21.69	5	4.34	30.64	0.0009
Pure Error	0.71	5	0.14		
Cor Total	280.53	19			

All the factors and interactions selected by the quadratic model were included in the equation (unreduced equation) even if the *p*-value for temperature, temperature-pH interaction, and temperature-time interaction was higher than 0.05. The importance of the variables and their effects on the production could be elucidated by the magnitude and the sign of regression coefficient of the actual or coded values generated by Design-Expert. Positive coefficients indicate a linear effect on the responses while negative coefficients reveal the opposite influence [42]. The higher magnitude influences the yield strongly to reduce or maximize it depending on the sign of the coefficients.

Interactions among temperature, pH and time.

**Fig. 4:**
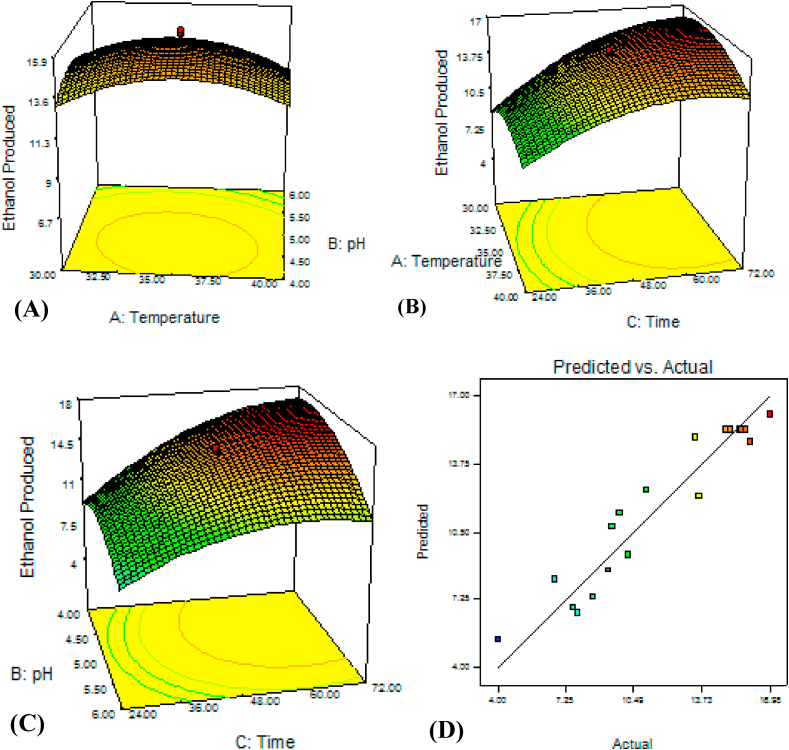
*K. marxianus* ETP87's response surface and contour plate of temperature vs. pH (A), temperature vs. time (B), pH vs. time (C), and correlation plot showing the distribution of actual (observed) and predicted values of ethanol produced from 40 g/L.

The data showed that ethanol production did not significantly vary with interaction between pH and temperature between 30 and 40 °C (Fig. 4 A) which was the optimal temperature range for ethanol yield. In a single factor (temperature study alone), *K. marxianus* ETP87 grew up to 50 °C with significant reduction in ethanol production after 45 °C, and was able to produce higher ethanol at 30–35 °C [43], 37 °C [24] and 40 °C [44]. The yeast grew and yielded best at pH lower than 5.5. Since the yeast was isolated from acidic *ergo* (pH, 3.7); it could adapt to the lower pH. Similarly in other studies, the optimal ethanol was produced by *K. marxianus* at pH 5.05 [44] and 4.8 [45].

The interaction between temperature and incubation time showed that optimal ethanol production was detected at higher than 40 h and in nearly all temperature range (Fig. 4 B). Unlike *P. fermentans* ETP22 and *S. cerevisiae* ETP53, optimal location was not at the center when pH was interacted with time (Fig. 4C). The optimum production was attained at pH lower than 5.5 and incubation time higher than 48 h. In a single factor study, maximum ethanol yield was obtained within 75 h of incubation using thermotolerant *K marxianus* IMB3 under batch condition [39]. The interaction of temperature (34.42 °C), pH (4.24) and incubation time (71.93) was able to produce optimal ethanol (17.22 g/L).

The adjusted R-squared value was 0.8851which indicated that the variations in ethanol production were contributed by the three factors at 88.51% confidence level hours) resulted maximum ethanol (17.22 g/L).

## 4 *P. fermentans* ETP22

Table 9 shows the combination of three interacting variables with actual and predicted value randomized by Expert Design. The predicted value was strongly correlated with actual value; therefore, the model was fit to predict by employing independent variables.

**Table 9:** Central composite design matrix for three independent variable with actual and predicted values of ethanol produced from 40 g/L dextrose by *P. fermentans* ETP22.

Standard order	Run order	Temperature (^o^C)	pH	Time (hours)	Actual value (g/L)	Predicted value (g/L)	Residual
1	5	30	4	24	6.72	5.38	1.34
2	16	40	4	24	4.18	3.00	1.18
3	9	30	6	24	5.76	4.52	1.24
4	19	40	6	24	0.22	0.07	0.15
5	15	30	4	72	9.23	8.19	1.04
6	18	40	4	72	0.42	0.46	−0.04
7	7	30	6	72	10.52	10.50	0.02
8	2	40	6	72	0.55	0.70	−0.15
9	10	26.59	5	48	8.78	10.37	−1.59
10	3	43.41	5	48	0.02	0.12	−0.10
11	20	35	3.32	48	2.73	4.25	−1.52
12	8	35	6.68	48	3.55	3.72	−0.17
13	12	35	5	7.64	0.6	2.35	−1.75
14	11	35	5	88.36	5.29	5.23	0.06
15	13	35	5	48	8.81	8.25	0.56
16	6	35	5	48	8.02	8.25	−0.23
17	14	35	5	48	7.75	8.25	−0.50
18	17	35	5	48	8.64	8.25	0.39
19	4	35	5	48	7.91	8.25	−0.34
20	1	35	5	48	8.67	8.25	0.42

The experimental responses (ethanol produced, g/L) were analyzed using ANOVA to estimate the impact of temperature, pH and time. The F-value analysis in ANOVA table exhibits that temperature had the highest impact whereas pH had the lowest contribution (Table 10). In fit summary analysis, the quadratic model was suggested by the software (Design-Expert). The empirical model in terms of actual factors is given as

Equation 6 $$\text{Y}=-96.48+3.24{\text{X}}_{1}+16.99{\text{X}}_{2}+0.52{\text{X}}_{3}-0.1{\text{X}}_{1}{\text{X}}_{2}-0.01{\text{X}}_{1}{\text{X}}_{3}+0.03{\text{X}}_{2}{\text{X}}_{3}-0.04{\text{X}}_{1}^{2}-1.51{\text{X}}_{2}^{2}-2.74{\text{X}}_{3}^{2}$$

where Y was the ethanol produced (g/L) and X_1_, X_2_, and X_3_ were temperature, pH and incubation time respectively.

**Table 10:** ANOVA for response surface (temperature, pH and time) quadratic model of P. *fermentans* ETP22.

Source	Sum of Squares	Df	Mean Square	F Value	p-value Prob > F
Model	230.06	9	25.56	17.26	<0.0001
A-Temperature	126.67	1	126.67	85.54	<0.0001
B-pH	0.33	1	0.33	0.22	0.6473
C-Time	10.07	1	10.07	6.80	0.0261
AB	2.16	1	2.16	1.46	0.2546
AC	14.31	1	14.31	9.66	0.0111
BC	5.02	1	5.02	3.39	0.0953
A^2^	16.29	1	16.29	11.00	0.0078
B^2^	32.80	1	32.80	22.15	0.0008
C^2^	35.86	1	35.86	24.22	0.0006
Residual	14.81	10	1.48		
Lack of Fit	13.76	5	2.75	13.16	0.0067
Pure Error	1.05	5	0.21		
Cor Total	244.87	19			

Though the *p*-value for pH, temperature-pH, and pH-time was higher than 0.05; all factors and interactions were considered in the equation. The *p*-value of the model was 0.0001 and this value intensified the significance of the model. A smaller *p*-value inferred a significant influence to the interaction than the higher one.

Interaction among temperature, pH and time.

**Fig. 5:**
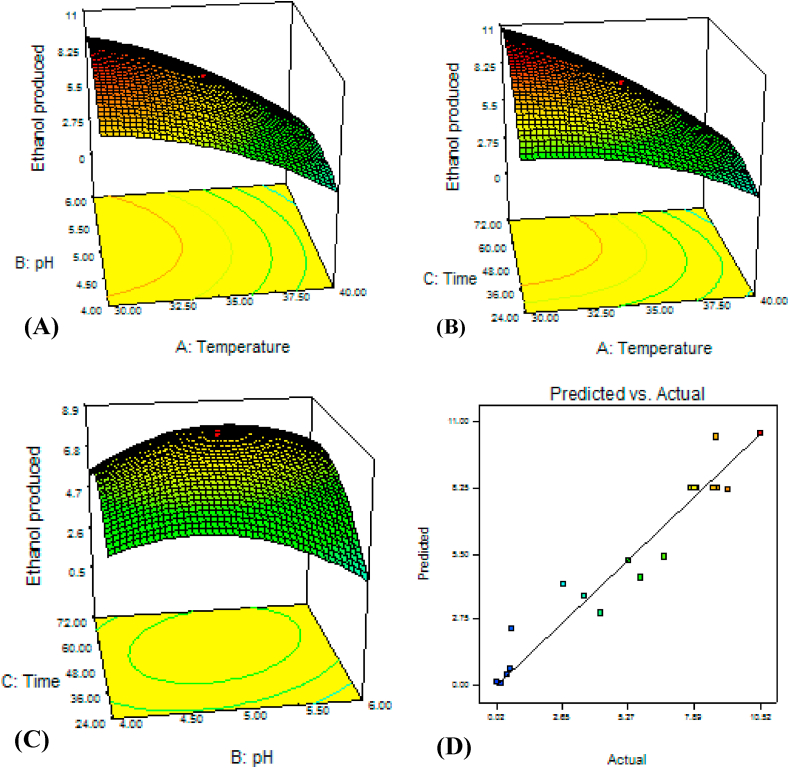
*P. fermentans* ETP22's response surface and contour plate of temperature vs. pH (A), temperature vs. time (B), pH vs. time (C), and correlation plot showing the distribution of actual (observed) and predicted values of ethanol produced from 40 g/L.

The interaction effect of temperature, pH and time on the ethanol yield from dextrose was illustrated graphically by plotting the three dimensional response surfaces and the two dimensional isoresponse contour (Fig. 5). The optimum ethanol production was achieved at higher incubation time (61.3 h), mild acidic pH (5.4), and lower temperature (30.3 °C). The ethanol yield was significantly reduced at the temperature higher than 34 °C (Fig. 5 A and B). pH-time interaction was weaker than temperature-pH and temperature-time interaction to maximize ethanol titer.

[46] showed that the optimal ethanol yield (10.86 g/L) was produced by *Pichia fermentans* at 30.39 °C, pH 5.13, and 59.38 h with unreduced model. *P. fermentans*, was able to produce 13 g/L ethanol at pH 6.5 and temperature of 30 °C [46]. [47] reported a production of 55 g/L ethanol by *P. fermentans* NBRC 1164 using medium that contained high dextrose (150 g/L) and corn steep liquor under aerobic condition and at 30 °C within 24 h.

## 5 Conclusions

This study revealed that the local strains of *S. cerevisiae* ETP53, *K. marxianus* ETP87, and *P. fermentans* ETP22 showed similar trend in ethanol production to the commercial baker's yeasts. The high survival rate (40–70%) of the non-*Saccharomyces* yeasts was similar to the *S. cerevisiae* strain, under 10% ethanol shock treatment which is a desirable characteristic of high fermenting yeasts. Their ability to grow and ferment different monosaccharaides (5-carbon, 6-carbon sugars), and disaccharides could make them good candidates for ethanol production from different carbon sources, whey, and lignocellulosic hydrolysates. It is particularly interesting to note that the non-Saccharomyces yeast, *K. marxianus* ETP87 utilized and vigorously fermented more sugars than even *Saccharomyces cerevisiae* strain ETP53, and was as equally efficient in ethanol production as the latter, and the only isolate that grew and vigorously ferment lactose could be singled out for further studies under optimum conditions.

Optimal ethanol was produced by *S. cerevisiae* ETP53 at 100 g/L dextrose with 5.5$\times 10^{6}$ inoculum size. The sugar content of the media sharply declined for the first 10 h incubation time suggesting that fast sugar absorption and utilization by the three isolates. The optimum pH, temperature, and time for *S. cerevisiae* ETP53, *K. marxianus* ETP87 and *P. fermentans* ETP22 were 4.5–5.5, 28–30, and 48–76; 3.8–5.2, 35–40 and 48–76; and 4.5–6.0, 30–32, 48–80, respectively. The optimization, in this study, was performed using YPD. It is more appropriate if it is going to be done in the actual media in which ethanol is produced in industry.
